# The Operative Treatment of Carcinoma Mammæ

**Published:** 1909-04-03

**Authors:** 


### Surgery.

### THE OPERATIVE TREATMENT OF CARCINOMA MAMMÆ.

The so-called complete operation for carcinoma mammæ, and by this we mean the complete removal of the breast with the pectoral muscles and the axillary lymphatic glands, first advocated by Halstead, has now been employed for a sufficient length of time to enable us to compare the results obtained by its means with those resulting from less radical measures. The history of the surgical treatment of carcinoma mammæ is interesting. It is not so very long since surgeons regarded suppuration in the wound as tending to reduce the liability to recurrence, and they used to fill the wound with dried peas with this object, the argument being that a tremendous reaction was produced which was sufficient to kill any portion of cancer that was left behind. Then the pendulum swung to the opposite extreme with the introduction of antiseptic methods, and primary union of the wound was then sought for with equal avidity. In the desire for this incisions were made which did not skirt the growth sufficiently widely so as to avoid tension when the wound was sewn up. Infected tissue was very often left behind, and recurrence was common, so common, in fact, that many surgeons regarded the operation as unjustifiable.

This led to the proposal to remove the axillary glands in all cases of carcinoma, a question which produced much controversy: the argument of the older school being that the glands acted as a filter which would at any rate prevent the general dissemination of the disease, and that it was therefore an unjustifiable proceeding unless the glands were felt to be definitely enlarged and involved. But there was a complete answer to this which has been amply supported by subsequent experience, that even for an expert surgeon it is not possible to say by palpation whether the glands are involved or not. In fact, one may go further and say that even if they are removed it is often impossible to give a definite opinion on the macroscopical appearances alone; a gland which is hardly, if at all, enlarged being often found histologically to be extensively infected. And even if they are not involved at the time of the first operation, they will subsequently become so, and a second operation will be necessary.

Eventually those who advocated removal of the axillary glands won the day, but it was left for Halstead to point out that to be consistent all possible sources of lymphatic infection should be removed, and that the most important of these is the lymphatic system in the fascia covering the pectoral muscle, and that the best way of getting rid of it is to remove the whole muscle. Thus was evolved the complete operation for carcinoma mammæ.

An incision is made from a point external to the tip of the coracoid process. From this point it descends and then divides, skirting the growth widely, and the two incisions meet again below the breast. The skin flaps so formed are dissected up thoroughly. The pectoralis major and minor are then freed from their thoracic attachments, and the breast, together with the muscles, is dissected up. The muscles are cut transversely near their insertion, and this gives a good exposure of the axillary tissues, particularly the axillary artery and vein. The latter is an important point, because when the surgeon knows exactly where these structures lie he can remove the glands with greater freedom. The axilla should then be dissected out, and removed in one piece with the breast without cutting across any lymphatic vessels if possible. Infection of the wound with cancer cells is thus avoided. The raw area so exposed is a large one, and it is therefore well before sewing up to put in a drainage tube through a separate opening in the posterior skin flap. The wound is then sewn up.

There are one or two small practical points in the operation and in the after treatment. The upper end of the incision should not be made too far out, otherwise when the wound heals the upper part of the scar will be in the axilla itself, and this may impede movements of the arm. When the operation was first performed it was the custom to bind the patient's arm to the side during convalescence. It was then found that great disability resulted from stiffness and adhesions. To overcome this the arm was kept at right angles to the trunk after the operation until the wound was healed. But a still better way is to leave it entirely free, so that the patient may use it herself from the beginning. If this is done, surprisingly little loss of function results considering the extent of the operation.

This is the best surgical treatment for carcinoma mammæ as far as we know to-day; but it is not applicable to all cases. Some, unfortunately, are allowed to progress to such a degree that the radical operation is neither possible nor justifiable. In such cases, if any operation is done at all, it must be a palliative one—*i.e.* it is performed to relieve the patient of the local effects of a fungating mass, and not with any hope of effecting a cure.

The principal points which render the complete operation unjustifiable are (1) fixation of the growth through the pectoral muscles to the thoracic wall; (2) metastatic deposits in the viscera or bony system, and (3) advanced cachexia. But in most cases it is justifiable to do a palliative operation—*i.e.* to remove the growth locally by an elliptical incision; for if it is not already fungating, it will do so sooner or later, and the possession of such a mass will render the patient's life a misery. If, however, the skin is so extensively involved that great tension will be necessary to bring the edges of skin together, or if it is likely that a raw area will be left it is better to avoid operating altogether. For if an infected area of thoracic wall is left exposed in the wound it grows with astonishing rapidity, so that in a few weeks there is a sprouting mass almost as big as the original tumour.

In order, therefore, to get the best possible results with carcinoma of the breast it is essential to operate early as soon as the diagnosis has been made, and to operate radically.

